# A feasibility study of combined epigenetic and vaccine therapy in advanced colorectal cancer with pharmacodynamic endpoint

**DOI:** 10.1186/s13148-021-01014-8

**Published:** 2021-02-02

**Authors:** Katherine M. Bever, Dwayne L. Thomas, Jiajia Zhang, Ernie A. Diaz Rivera, Gary L. Rosner, Qingfeng Zhu, Julie M. Nauroth, Brian Christmas, Elizabeth D. Thompson, Robert A. Anders, Carol Judkins, Meizheng Liu, Elizabeth M. Jaffee, Nita Ahuja, Lei Zheng, Nilofer S. Azad

**Affiliations:** 1 Department of Oncology, Sidney Kimmel Comprehensive Cancer Center, Bloomberg~Kimmel Institute for Cancer Immunotherapy, Johns Hopkins University School of Medicine, 1650 Orleans Street, Office 4M10, Baltimore, MD 21287 USA; 2 Department of Pathology, Johns Hopkins University School of Medicine, Baltimore, MD USA; 3 Division of Biostatistics and Bioinformatics, Sidney Kimmel Comprehensive Cancer Center, Johns Hopkins University School of Medicine, Baltimore, MD USA; 4 Department of Surgery, Johns Hopkins University School of Medicine, Baltimore, MD USA; 5 Departments of Surgery, Oncology, and Pathology, Smilow Comprehensive Cancer Center, Yale University School of Medicine, New Haven, CT USA

**Keywords:** GVAX, Immunotherapy, Epigenetic therapy, Colorectal cancer

## Abstract

Epigenetic therapies may modulate the tumor microenvironment. We evaluated the safety and optimal sequence of combination DNA methyltransferase inhibitor guadecitabine with a granulocyte macrophage-colony-stimulating-factor (GM-CSF) secreting colon cancer (CRC) vaccine (GVAX) using a primary endpoint of change in CD45RO + T cells. 18 patients with advanced CRC enrolled, 11 underwent paired biopsies and were evaluable for the primary endpoint. No significant increase in CD45RO + cells was noted. Grade 3–4 toxicities were expected and manageable. Guadecitabine + GVAX was tolerable but demonstrated no significant immunologic activity in CRC. We report a novel trial design to efficiently evaluate investigational therapies with a primary pharmacodynamic endpoint.

*Trial registry* Clinicaltrials.gov: NCT01966289. Registered 21 October, 2013.

## Background

Despite improvements over the past several decades in the management of colorectal cancer (CRC), unresectable advanced disease remains largely incurable with responses to currently available systemic treatments of limited duration. Immunotherapy has the potential to induce deep and durable responses even in advanced treatment-refractory cancers; however, thus far the activity of immunotherapy in CRC has been limited to those that have mismatch repair deficiency (dMMR), which represents less than 5% of advanced CRC cases [1]. Response to immunotherapy remains elusive in the 95% of patients with proficient MMR/microsatellite stable CRC, despite accumulating evidence that an anti-tumor immune response plays a significant role in prognosis regardless of MSI/MMR status [2, 3]. Novel immunotherapy combinations may be needed to further modulate the immune microenvironment to achieve an effective anti-tumor immune response in these prototypically “cold” tumors.

Epigenetic therapy has recently emerged for its potential role in such combinations. Epigenetic alterations govern the differential expression of genetic information and have a prominent role in cancer initiation and progression [4]. Agents targeting the epigenome include DNA methyltransferase 1 inhibitors (DNMTi) and histone deacetylase inhibitors (HDACi). While anti-tumor activity of these agents has been observed in hematologic malignancies, in solid tumors including CRC, trials of epigenetic therapy have failed to demonstrate an effect of these agents alone and treatment is often limited by toxicities [5–9]. However, multiple preclinical studies have demonstrated modulation of the tumor immune microenvironment by these epigenetic therapies through a number of mechanisms, including increased expression of HLA Class I antigens, cancer/testis antigens and genes related to antigen presentation, upregulation of interferon-gamma receptor and signaling proteins, and upregulation of immune checkpoint expression [10–17]. These data suggest a potential role for epigenetic therapy in sensitizing the tumor to immunotherapy.

Guadecitabine is a second-generation DNMTi with enhanced resistance to cytidine deaminase inactivation when compared to first-generation agents in this class, resulting in a longer half-life and greater systemic exposures [18]. Guadecitabine has been shown in preclinical models to have a potent immunomodulatory effect through the increased expression of cancer/testis antigens and HLA class I antigens, strongly supporting a potential role in combinatorial approaches [19].

We hypothesized that the addition of guadecitabine to a GM-CSF secreting colon vaccine (GVAX) would enhance the anti-tumor immune response and thus trafficking of effector T cells into the tumor. The GVAX colon cancer vaccine consists of two allogeneic colon cancer cell lines combined with a bystander cell line modified to secrete GM-CSF as a vaccine adjuvant. A whole cell vaccine has the advantage of delivering a range of peptide antigens which may be shared between the vaccine cell lines and the patient’s tumor. GVAX (given with immunomodulatory doses of cyclophosphamide (Cy) to deplete regulatory T cells in the tumor microenvironment) has been shown to be safe and induced immune responses albeit in the absence of clinical responses [20, 21]. In a mouse model, the combination of Cy/GVAX with the DNMTi decitabine showed promise; however, sequence of administration appeared to be important. Co-administration of decitabine with Cy/GVAX inhibited tumor growth and was associated with enhanced interferon gamma production both in splenocytes and tumor infiltrating lymphocytes (TILs), while decitabine administration after Cy/GVAX resulted in increased tumor growth, likely through inhibition of the immune response[22].

We further hypothesized that an increase in TILs, specifically CD45RO+ cells, on sequential tumor biopsies could be used as a surrogate for clinical outcomes and would therefore enable the more efficient evaluation of the regimen. CD45RO+ memory T cell density was demonstrated across several studies to have prognostic significance both in early and advanced cancers including colorectal cancer and were felt to broadly represent the T cell compartment[23–25].

We initiated a clinical trial to test optimal sequence of guadecitabine in combination with the colon GVAX utilizing a primary biological endpoint of change in CD45RO+ TILs.

## Patients and methods

### Patient selection

Patients were eligible for participation in this study if they were 18 years of age or older and had histologically proven adenocarcinoma of the colon or rectum that was treated with at least 1 prior systemic therapy for metastatic disease. Disease amenable to biopsy with acceptable clinical risk was required for participation. Other key inclusion criteria included Eastern Cooperative Oncology Group Performance Status of 0 or 1, life expectancy of greater than 4 months, and adequate organ and marrow function. Patients were excluded who had a known history or evidence of brain metastases, prior treatment with demethylating agents, autoimmune disease, uncontrolled intercurrent illness, or other contraindications to receive the study drugs or their components.

### Study design and endpoints

**Fig. 1:**
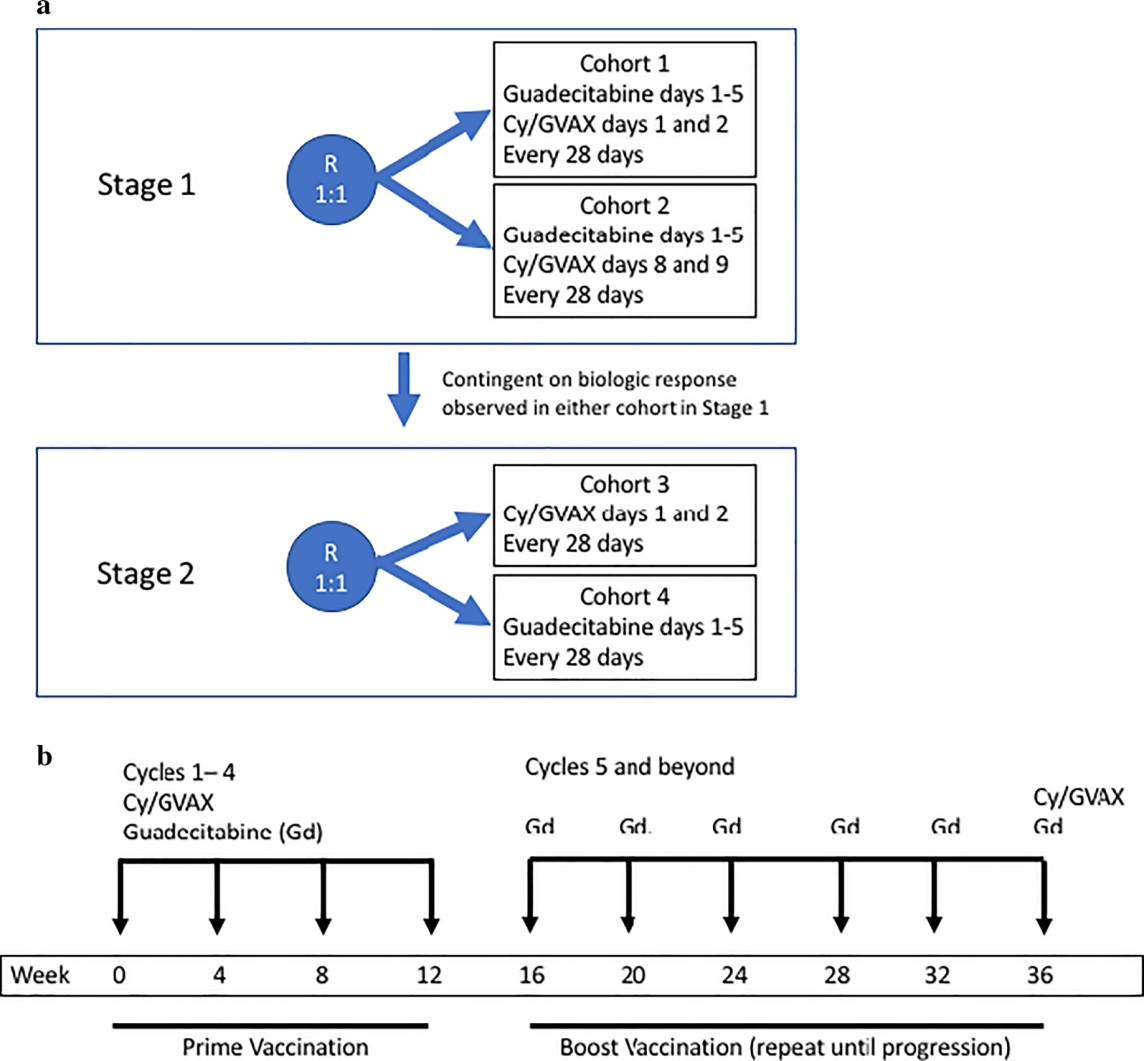
**a** Overall study design. **b** Treatment schema for Stage 1

This was a single-center, open label, randomized pilot study designed to evaluate the effect of Guadecitabine and Cy/GVAX using an immune endpoint of change in CD45RO+ TILs on pre- and post-treatment biopsies. The study was designed to be conducted in two stages (Fig. 1a). In stage 1 of the trial, patients were randomized in a one-to-one ratio either to receive Cy/GVAX concurrently with guadecitabine (Cohort 1) or to receive Cy/GVAX after guadecitabine (Cohort 2). A second stage was planned contingent on an observed effect in at least one of the cohorts in Stage 1. In Stage 2, patients would be randomized to either Cy/GVAX or guadecitabine alone, to evaluate the immunologic response to the individual components and select the optimal sequence of therapy.

The study protocol was approved by the institutional review board of the Johns Hopkins School of Medicine and was conducted according to the Declaration of Helsinki and the guidelines for Good Clinical Practice. All patients signed a written informed consent before the conduct of any study procedures and after a full explanation of the study to the patient by the study investigator.

The primary objectives of the study were to evaluate changes in TILs by histology in pre- and post-treatment tumor specimens in mCRC patients treated with guadecitabine and cyclophosphamide/GVAX, and to determine the safety and feasibility of the combination. Secondary objectives were to determine the optimal sequence of the combination using changes in immune endpoints, and to estimate overall survival, time to progression, and progression-free survival in patients with metastatic colorectal cancer treated with the combination. Exploratory endpoints included analysis of baseline and post-treatment tumor specimens for other immune endpoints.

### Study procedures

Treatment was administered on 28-day cycles and consisted of guadecitabine 45 mg/m^2^ administered subcutaneously on days 1–5, and Cy and GVAX either on days 1 and 2 respectively (Cohort 1) or days 8 and 9 (Cohort 2) (Fig. 1b). Cyclophosphamide 200 mg/m2 was delivered intravenously on the day prior to GVAX treatment, and GVAX consisted of 8–9 intradermal injections for a final total dose of 5 × 10^8^ colon cancer cells plus 5 × 10^7^ GM-CSF secreting cells. If patients demonstrated evidence of disease response or stabilization after 4 cycles of therapy, they were eligible to continue therapy with guadecitabine monthly and boost vaccinations every 6 months in the sequence in which they were administered in their previously assigned cohort. Tumor samples were collected by core biopsy at baseline and on day 15 of cycle 2.

Adverse events were recorded and graded according to the National Cancer Institute Common Terminology Criteria for Adverse Events Version 4.0. Events classified as possibly, probably, or definitely related by the study investigator were considered treatment-related. Radiographic assessments of response were performed every 8 weeks and analyzed for response according to Response Evaluation Criteria in Solid Tumors (RECIST) version 1.1[26]

### Immunohistochemistry

Tumor core biopsies specimens were obtained prior to and after treatment in order to evaluate the TILs in the tumor microenvironment. The core biopsies were formalin-fixed paraffin-embedded (FFPE) and stored in -20 °C. Immunohistochemistry (IHC) staining was performed on sequential 5 μm FFPE sections. Immunostaining for CD45RO was performed at the Johns Hopkins Hospital Immunopathology Laboratory using the Ventana autostainer with mouse monoclonal anti-CD45RO (MB-1) antibody (Ventana Medical Systems, Inc., Tucson, AZ, USA) according to the manufacturer’s protocols. CD8 IHC was completed at the Johns Hopkins University School of Medicine Tumor Microenvironment core utilizing mouse monoclonal anti-CD8 (4B11) antibody using the automated Leica Bond RX platform. All IHC was performed with 3–3′-diaminobenzidine detection and haematoxylin counterstaining. Manual multiplex IHC (mIHC) was performed as previously described [27].

Manual immunostaining for PD-L1 was completed as previously described28. The tissue specimens were deparrafinized, rehydrated, and immediately stained with the PD-L1 antibody clone SP142 (Spring Bioscience). Detection was performed by Tyramide Signal Amplification (TSA) system (PerkinElmer).

### Image analysis

All slides were anonymized, scanned using Aperio ScanScope CS and Hamamatsu XR (NanoZoomer), and whole-slide images were analyzed using HALO Image Analysis Platform (Indica Labs, Corrales, NM). Tumor area for analysis was determined by trained pathologists (EDT and RAA). For CD45RO, the membranous algorithm was used. For CD4, CD8, CD68, and CD163, the immune cell algorithm was used.

### Statistical analysis

A sample size for the biological endpoint was chosen targeting a true immune response rate of 40%. A significant immune response was defined as an increase in CD45RO+ cells from baseline of at least two-fold and 1.8 times the standard deviation of baseline samples calculated across patients. A two-stage design was employed, whereby enrollment would continue until 5 evaluable subjects were enrolled in each cohort. If 1 out of the first 5 evaluable subjects demonstrated an immune response, the cohort would be expanded to 8 subjects. If at least 2 out of 5 (or 8) subjects exhibited an immune response, the regimen would be considered promising, and if 0 out of 5 or 1 out of 8 had an immune response, the regimen would be deemed not worthy of further exploration. This design gave an 87% power to detect a true 40% immune response rate across patients with a one-sided exact type I error rate of 0.057 calculated from the binomial distribution.

Analysis of safety and efficacy endpoints were performed on all patients who received at least one dose of study drug. Adverse events and toxicity were classified and graded according to the Common Toxicity Criteria for Adverse Events (CTCAE) version 4.0. Kaplan–Meier curves were used to estimate probabilities of progression-free survival and overall survival, and univariable Cox proportional-hazards model was used to compare differences in time to progression and overall survival between two treatment groups. All statistical tests were two-sided, and p-values < 0.05 were considered significant. All statistical analyses were performed using R version 3.5.3.

## Results

### Patients and treatment

Between April 2014 and November 2016, 18 patients were enrolled and randomized (Cohort 1 = 8 patients, Cohort 2 = 10 patients) to achieve the minimum of 5 paired biopsies per arm; in total 11 patients underwent paired biopsies and were therefore evaluable for the primary endpoint. One patient in Cohort 1 did not receive treatment. Enrollment was terminated in Stage 1 due to slow accrual. Demographics and baseline disease characteristics are presented in Table 1. Patients were heavily pretreated with a median of 2 (range 1–7) prior therapies in the metastatic setting.

**Table 1:** Baseline patient characteristics

Characteristic	N = 18
*On-study age—years*	
Median	57
Range	25—81
*Sex-no. (%)*	
Female	10 (56)
Male	8 (44)
*Ethnicity-no. (%)*	
Non-hispanic white	7 (39)
Non-hispanic African-American	6 (33)
Other	5 (28)
Primary site (%)*	
Left colon/rectum	11 (61)
Right colon	6 (33)
*Histologic grade*	
Well differentiated	0 (0)
Moderately differentiated	14 (78)
Poorly differentiated	2 (11)
Unknown	2 (11)
*Time from metastatic diagnosis-months*	
Median	32
Range	4—172
*Metastatic site (%)*	
Liver only	4
Lymph nodes only	1
Bladder only	1
Multiple sites	12
*Exposure to prior therapies (%)*	
Fluoropyrimidine	18 (94)
Oxaliplatin	17 (94)
Irinotecan	17 (94)
Anti-VEGF	17 (94)
Anti-EGFR	8 (44)
*KRAS mutation status*	
Wild-type	7
Mutant	9
Unknown	2
*Microsatellite Instability/Mismatch Repair Status*	
MSI-H/dMMR	1
MSS/pMMR	12
Unknown	5

*Unable to verify site of primary in 1 patient

### Biological endpoint

**Fig. 2:**
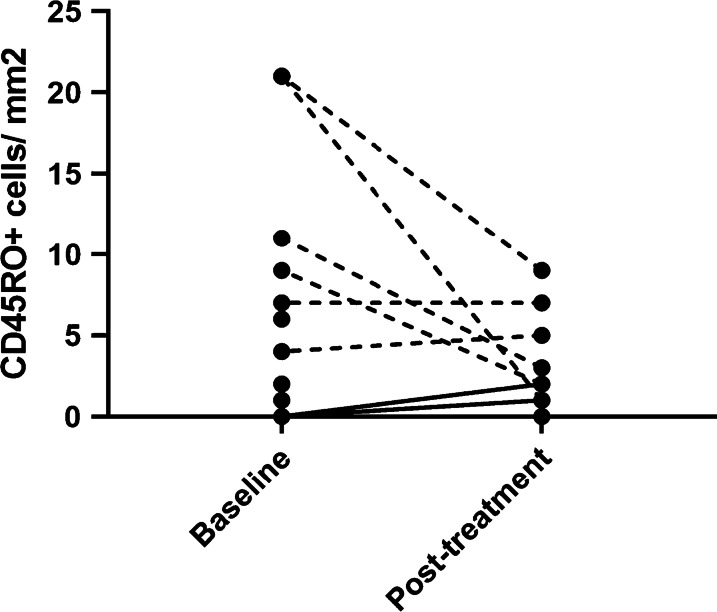
Plot of CD45RO-positive cells per mm2 of tumor area as quantified by immunohistochemistry. Matched baseline and post-treatment measurements are connected by a line. In total, 17 samples were obtained at baseline and of those, 11 had matched post-treatment biopsies. 3 patients had 0 positive cells both at baseline and post-treatment, and 2 patients had 0 positive cells at baseline and no paired post-treatment biopsy. The two patients with change from absent infiltrate at baseline to mild to moderate infiltrate on treatment are indicated by a solid line. *Note: A very high pre-treatment measurement (125 CD45RO+ cells/mm2 at baseline) is excluded from this figure; the patient had no corresponding post-treatment sample

A total of 17 patients underwent baseline tumor biopsies, and of those, 11 had matched pre- and post-treatment biopsies assessable for change in TIL density by CD45RO IHC. Results of computer-aided quantification are shown in Fig. 2. By this method, we did not observe any immunologic responses based on the prespecified minimum increase of CD45RO+ cells by two-fold and 1.8 times the standard deviation; however, in two patients on Cohort 1, an increase from no infiltrate at baseline to mild infiltrate (1 and 2 cells/mm2 respectively) was observed (solid lines), suggestive of a biological effect.

### Safety endpoint

Treatment-related adverse events are summarized in Table 2, and did not differ significantly by treatment arm. In general, treatment was well tolerated. No patients discontinued participation due to tolerability. The most frequently observed adverse events were vaccine and/or guadecitabine injection site reactions, one or both of which were observed in all patients. All of these events were mild (Grade 1–2) and self-limited. Treatment-related serious adverse events (Grade 3–4) occurred in 7 patients and were hematologic in nature (neutropenia and/or leukopenia) or fatigue. There were no treatment-related deaths.

**Table 2:** Treatment-related adverse events

Event-no.(%)	Cohort 1 (N = 7)		Cohort 2 (N = 10)	
	All grades	Grade 3 or 4	All grades	Grade 3 or 4
Any event	7 (100)	3 (43)	10 (100)	5 (50)
Injection Site Reactions**				
GVAX	7 (100)	0	8 (80)	0
Guadecitabine	5 (71)	0	7 (70)	0
*Generalized Symptoms*				
Fatigue	5 (71)	0	8 (80)	3 (30)
Fever/Chills	3 (43)	0	2 (20)	0
Myalgia	2 (29)	0	1 (10)	0
Headache	1 (14)	0	3 (30)	0
Flu-like illness	0	0	1 (10)	0
Dizziness	1 (14)	0	0	0
*Gastrointestinal*				
Constipation	2 (29)	0	2 (20)	0
Nausea/vomiting	3 (43)	0	2 (20)	0
Diarrhea	0	0	1 (10)	0
Flatulence	1 (14)	0	0	0
*Hematologic*				
Neutropenia	3 (43)	3 (43)	4 (40)	4 (40)
Leukopenia	0	0	1 (10)	1 (10)
Anemia	1 (14)	0	0	0
*Dermatologic*				
Rash/Pruritis	0	0	2 (20)	0
Hives	1 (14)	0	0	0
*Other*				
Cough	1 (14)	0	0	0
Warmth, thighs	1 (14)	0	0	0

*Events were counted once for each patient using the highest grading

**Includes erythema, induration, pruritis, hyperpigmentation, tenderness

### Clinical outcomes

The median time on treatment was 56 days (range 29–174 days). Three patients discontinued treatment prior to disease reassessment by imaging and therefore were not assessable for response endpoints. Of the remaining 15 patients, there were no objective responses to treatment by RECIST criteria. Two patients achieved a best response of stable disease, and 13 patients demonstrated progressive disease by the first scan. One patient in Cohort 2 completed 4 cycles of treatment followed by 2 boost doses. Median progression-free survival and overall survival for the entire cohort were 50 days (95% confidence interval (CI): 49–54) and 393 days (95% CI: 298 to 694), respectively. One patient from Cohort 1 remains alive as of the data cutoff in January 2020, having progressed on this trial and subsequently achieving a durable response to anti-PD-1 therapy on protocol for MSI-H disease.

### Correlative analysis

There was no correlation of change in CD45RO+ cells with clinical outcomes. Specifically, in the two patients with appreciable increase in TILs (from zero to positive infiltrate), there did not appear to be any clinical benefit. Among the four long-term survivors (surviving > 18 months from enrollment), two had complete absence of CD45RO+ TILs in both the baseline and post-treatment samples, including the MSI-H tumor. The other two long-term survivors had a moderate infiltrate (9 and 11 cells/mm2 respectively) at baseline; however, a decrease was noted in the on-treatment sample to 2 and 3 cells/mm2 respectively. Analysis of CD8+ cells by the same method yielded higher cell densities including in the MSI-H tumor; however, there was no appreciable trend with change on treatment. On the whole, neither baseline CD8+ cell density nor change with treatment appeared to correlate with outcomes.

Multiplex immunohistochemistry (mIHC) was performed to further characterize immune infiltrates in these tumors with multiple immunophenotypic markers. Analyses of changes in CD4+ , CD8+ , CD68+ and CD163+ cell densities between pre- and post-treatment samples are shown in Additional file 1: Figs. 1 through 4. While most immune subsets did not demonstrate a significant change with treatment, an increase in CD68+ :CD8+ cell density ratio for the total cohort was observed to be statistically significant (Additional file 1: Fig. 3). Importantly there was no observed trend in change of CD163+ :CD68+ cell density ratio. Furthermore, we observed an increase in CD4+ cells in response to treatment in the three long-term survivors with matched pre- and post-treatment biopsies.

IHC for PD-L1 was performed and proportion of tumor cells staining positive for PD-L1 was quantified as previously described[29]. In this limited data set, there was no obvious correlation with either score at baseline or change with treatment and outcomes (Additional file 1: Fig. 4). PD-L1 was also estimated semi-quantitatively. Six cases were negative for PD-L1 by this method at baseline; one of these patients’ tumor became PD-L1+ after therapy and this patient was among the long-term survivors.

## Discussion

Progress in the development of new therapies for advanced CRC has been slow, and in the era of burgeoning immunotherapeutics with innumerable potential combinations to be evaluated, large-scale phase III trials are impractical. Furthermore, few studies have addressed the optimal sequencing of the combinatorial approaches and novel study designs are needed to this end. We report the results of a trial of the combination of the DNMTi guadecitabine and GVAX colon cancer vaccine in patients with advanced colorectal cancer, the first trial, to our knowledge, designed to identify the optimal sequencing of an immunomodulatory combination using a primary biological endpoint. Our study utilized a primary endpoint of increase in CD45RO+ TILs, with an aim to characterize the best potential sequence of this combination prior to moving forward with well-powered phase II studies. The treatment was well tolerated with no unexpected toxicities. Based on the predefined endpoint of increase in CD45RO+ TILs no significant increase was noted, although an increase from no CD45RO+ T cell infiltrate to a measurable infiltrate was observed in two patients in Cohort 1, showing a possible biologic effect. Unfortunately, a clinical effect of the regimen was not appreciated, with most patients experiencing progressive disease by the first scan.

The design of our study permitted further investigation of effects on other immune cell populations in the tumor through the use of multiplex IHC analysis. In particular, effects of the agents under study on myeloid cell populations are poorly understood. GVAX has been shown to affect myeloid cell populations in a dose-dependent manner, with increased activation and numbers of circulating monocytes at lower doses and increased MDSCs at higher doses [30, 31]. In combination with a listeria based vaccine in metastatic pancreatic cancer, GVAX decreased CD68+ cells in the tumors of the subset of patients with longer survival [32]. Epigenetic alterations likely play a role in regulation of the macrophage phenotype; however, the different classes of agents may differentially impact the myeloid compartment. Specifically, HDACi therapy combinations have demonstrated pro-immune effects on monocyte-macrophage populations both in patients and in animal models, and effects on macrophage polarization are a postulated mechanism for the immunomodulatory effects of these agents [33–35]. DNMT1 positively regulates the M1 phenotype and some studies have demonstrated downregulation of M1 markers and increased expression of M2 markers with inhibition of DNMT1 [36, 37]; however studies of effects in tumor associated macrophages are lacking38. In this study, we observed a significant increase in the myeloid compartment with treatment (increase in CD68:CD8 ratio where CD8 density did not change with therapy); however, CD163:CD68 ratio did not change significantly with treatment, suggesting that the regimen did not promote polarization to the immune-suppressive M2 phenotype.

This study highlights some of the challenges and potential pitfalls of a small phase 0 study with a biological endpoint. Most importantly, the design relies on the validity of the primary biomarker. In this study and others of immunomodulatory agents, the optimal biomarker of response to immunotherapy is not known and remains a subject of intensive study and debate. Different immunotherapy approaches may each have unique single biomarkers or biomarker panels that are salient to the agent(s) being tested. We chose CD45RO+ cells due to multiple reasons, including prior reports that showed CD45RO+ T cell infiltration had prognostic significance and their underlying biology, representing the T cell compartment broadly [23–25]. However, whether change in CD45RO+ T cell infiltration has utility as a predictive marker has not been extensively validated. Several other potential biomarkers of response to immunotherapy have more recently emerged, including other immune cell markers, immune checkpoints (notably PD-L1) and gene expression signatures. These data are also limited, with most being in tumor types where responses to immune checkpoint inhibitors are commonly seen such as melanoma. It is not known to what extent these findings can be extrapolated to other tumor types and other immunotherapies. In this study there was no clear correlation between PD-L1 expression and clinical outcomes, utilizing two different methods for quantification of PD-L1.

Additionally, a biomarker based on core biopsies has several potential issues. Infiltration of immune cells within a tumor may be heterogeneous and not well represented with core needle biopsies [23]. The optimal means of quantification has not been established and computer-aided analysis has limitations. In addition, spatial information regarding distribution of TILs (at the invasive margin versus intratumoral) may be an important component of quantification and may be limited in biopsy cores [23]. Finally, paired biopsies are burdensome to patients, and difficult to obtain in all subjects. Analyses may be biased towards patients who are able to undergo a second biopsy. Thus the development of biomarkers based on newer non-invasive techniques such as liquid biopsy or imaging may be advantageous.

In summary, no significant clinical activity of guadecitabine with Cy/GVAX in metastatic colorectal cancer patients was observed in this small study. However, we report on a novel study design to evaluate optimal sequence of administration which could be carried forward in other studies of combinations. While we were not able to draw definitive conclusions regarding the immunologic activity of the regimen, it remains possible that other immune suppressive pathways are contributing to the lack of activity and further immune activation with another agent such as an immune checkpoint inhibitor may be necessary. The potential for epigenetic therapy to sensitize tumors to the activity of immunotherapy remains an area of interest, and other immunotherapy combinations continue to be explored [39].

## Supplementary information

**Additional file 1.** **Fig. 1:** Comparison of the cell density in **(A)** CD4^+^ cells (CD45^+^CD3^+^CD4^+^), **(B)** CD8+ cells (CD45^+^CD3^+^CD8^+^), **(C)** CD68+ cells, **(D)** CD163+ cells in pre- and post-treatment sections. NS, not significant, by unpaired t-test. **Fig. 2:** Multiplex immunohistochemistry staining for T-cell and macrophage markers on pre- and post-treatment biopsy sections, by survival. Upper panel (A-D) represents before-after plots for survival of less than 18 months. Comparison of the **(A)** cell density of CD4^+^ cells (CD45^+^CD3^+^CD4^+^), **(B)** CD8^+^ cells (CD45^+^CD3^+^CD8^+^), **(C)** CD68^+^ cells, **(D)** CD163^+^ cells in pre- and post-treatment biopsy sections. Lower panel (E–H) represents before-after plots for survival of greater than 18 months. Comparison of the **(E)** cell density of CD4^+^ cells (CD45^+^CD3^+^CD4^+^), **(F)** CD8^+^ cells (CD45^+^CD3^+^CD8^+^), **(C)** CD68^+^ cells, **(F)** CD163^+^ cells in pre- and post-treated biopsy sections. *P < 0.05. NS, not significant, by paired t-test. **Fig. 3:** Comparison of **(A)** CD68^+^ to CD8^+^ cell density ratio (cells/mm^2^ to cell/mm^2^) within the tumor area in pre- and post-treatment specimens. **(B)** CD163^+^ to CD68^+^ cell density ratio (cells/mm^2^ to cells/mm^2^) within the tumor area in pre- and post-treatment specimens. *P = NS, not significant, by unpaired t-test. **Fig. 4:** (A) Quantitative PD-L1 score at baseline plotted against survival. (B) Change in quantitative PD-L1 score from pre- to post-treatment plotted against survival.

## Data Availability

The datasets used and/or analyzed during the current study are available from the corresponding author upon reasonable request.
