# Supplementary figures and images for: A feasibility study of combined epigenetic and vaccine therapy in advanced colorectal cancer with pharmacodynamic endpoint
Source: Clin Epigenetics. 2021 Feb 2;13:25. doi: 10.1186/s13148-021-01014-8 (PMC7856736; doi:10.1186/s13148-021-01014-8)

**Supplemental Figure 1**

**A**

**B**

**C**

**D**


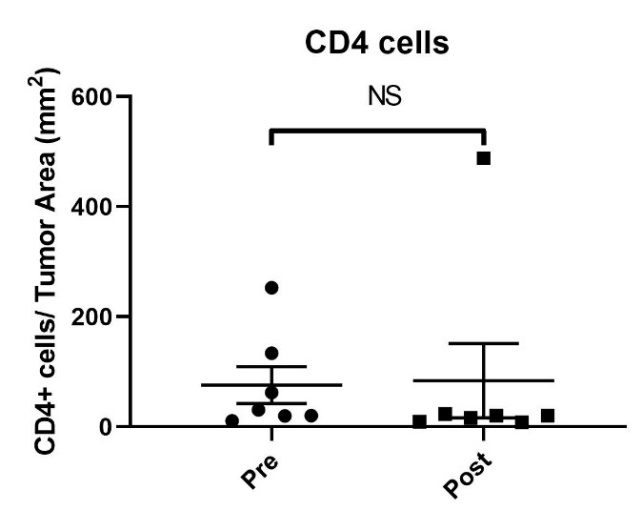

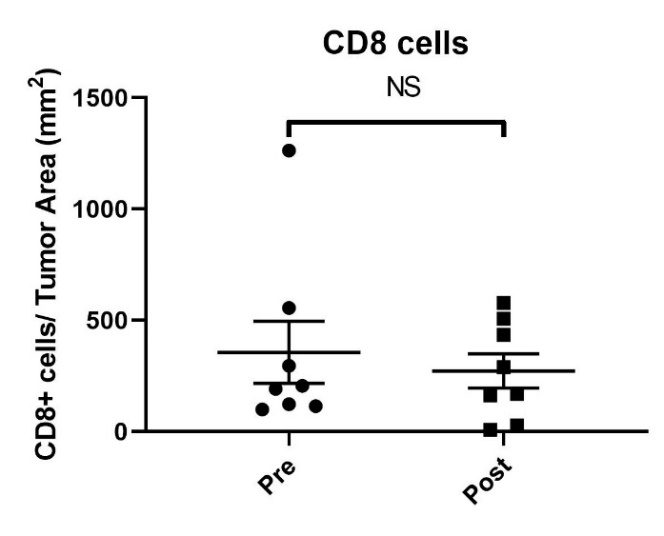

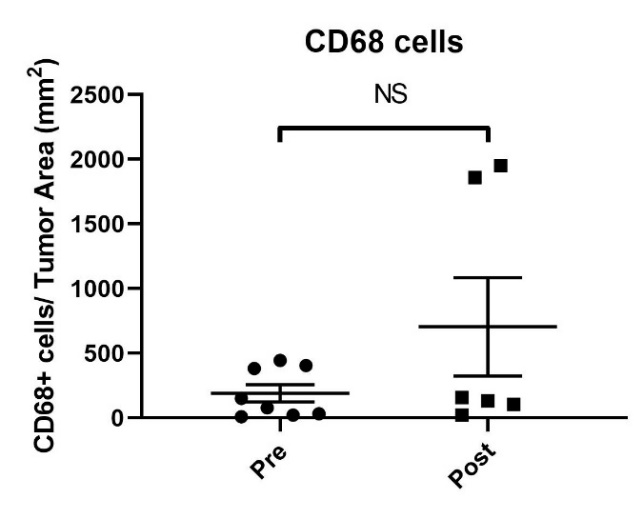

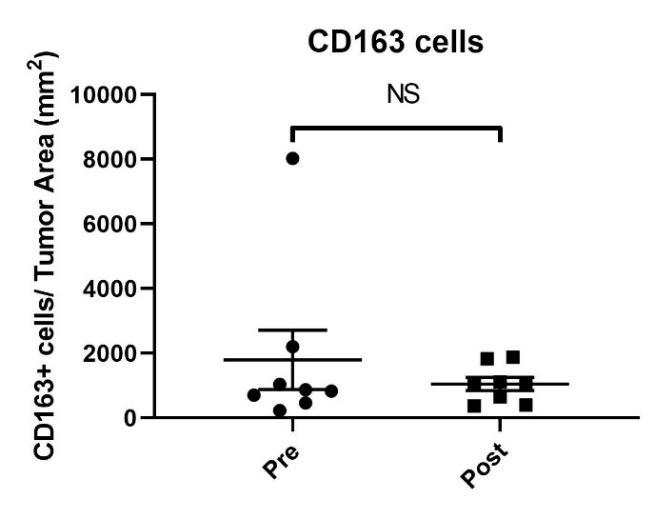


**Supplemental Figure 2.**


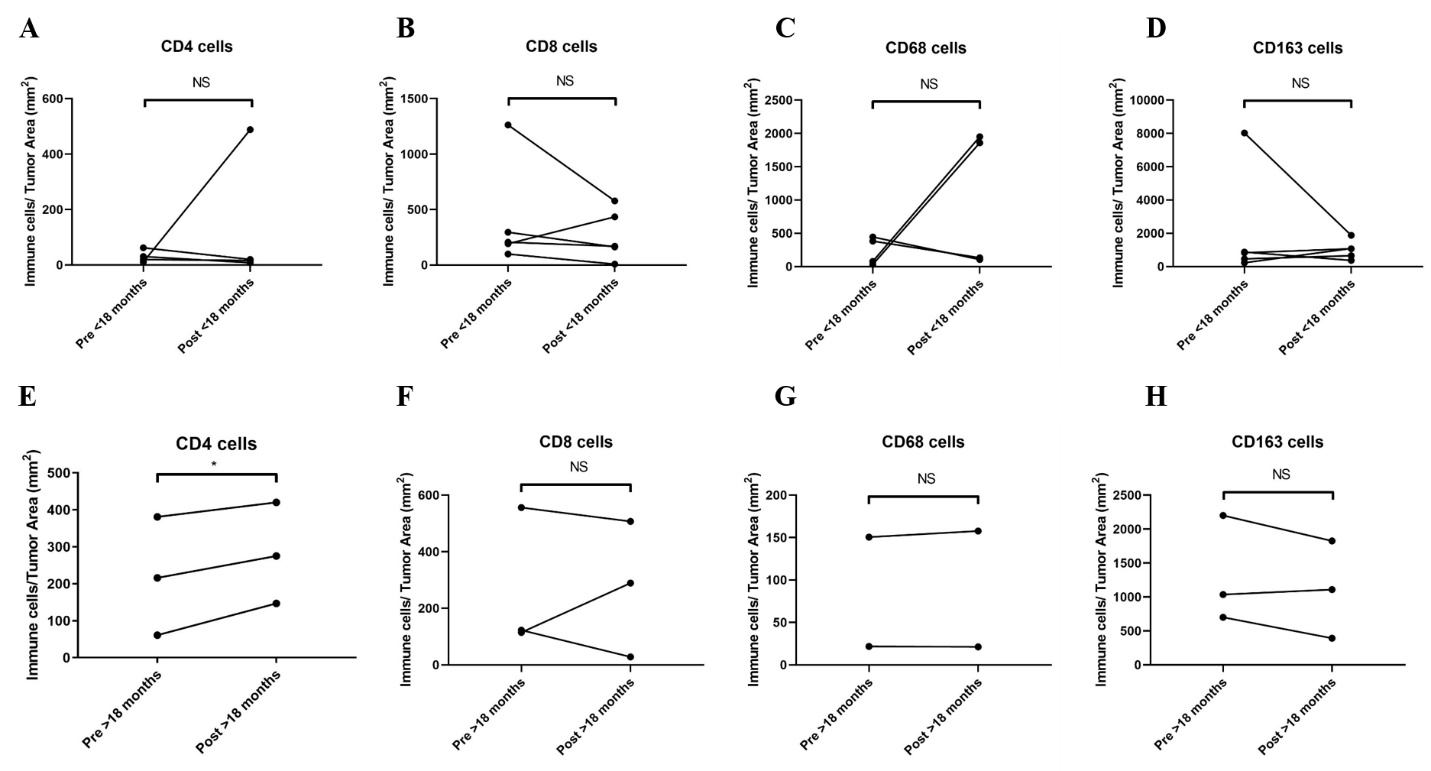


**Supplemental Figure 3**

**A**

**B**


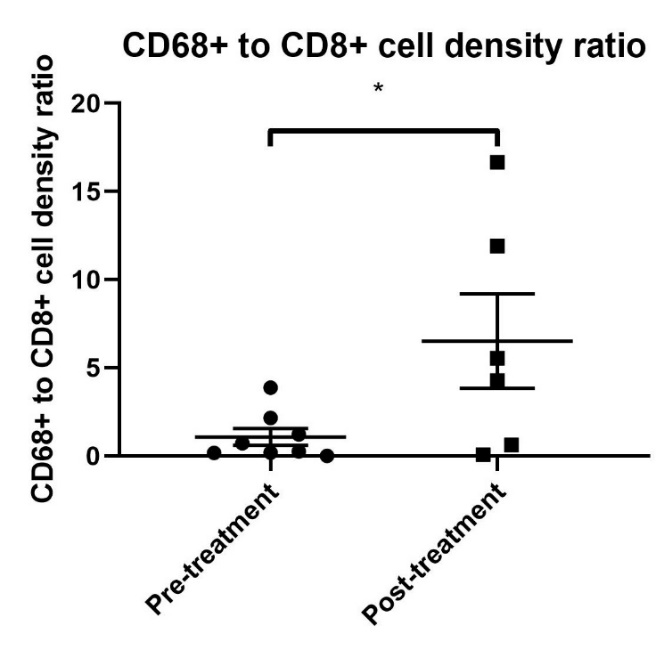

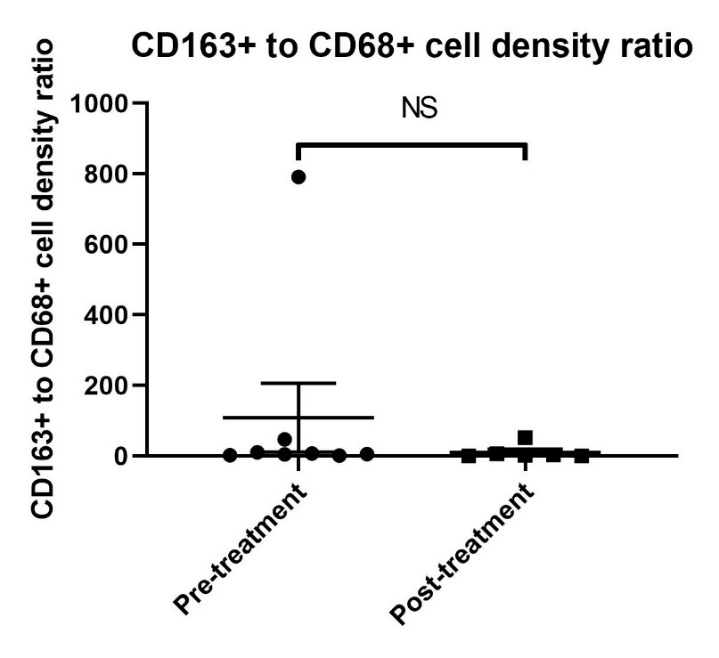


**Supplemental Figure 4**

Supplement: Supplementary file 1 — Additional file 1. Fig. 1: Comparison of the cell density in (A) CD4+ cells (CD45+CD3+CD4+), (B) CD8+ cells (CD45+CD3+CD8+), (C) CD68+ cells, (D) CD163+ cells in pre- and post-treatment sections. NS, not significant, by unpaired t-test. Fig. 2: Multiplex immunohistochemistry staining for T-cell and macrophage markers on pre- and post-treatment biopsy sections, by survival. Upper panel (A-D) represents before-after plots for survival of less than 18 months. Comparison of the (A) cell density of CD4+ cells (CD45+CD3+CD4+), (B) CD8+ cells (CD45+CD3+CD8+), (C) CD68+ cells, (D) CD163+ cells in pre- and post-treatment biopsy sections. Lower panel (E–H) represents before-after plots for survival of greater than 18 months. Comparison of the (E) cell density of CD4+ cells (CD45+CD3+CD4+), (F) CD8+ cells (CD45+CD3+CD8+), (C) CD68+ cells, (F) CD163+ cells in pre- and post-treated biopsy sections. *P < 0.05. NS, not significant, by paired t-test. Fig. 3: Comparison of (A) CD68+ to CD8+ cell density ratio (cells/mm2 to cell/mm2) within the tumor area in pre- and post-treatment specimens. (B) CD163+ to CD68+ cell density ratio (cells/mm2 to cells/mm2) within the tumor area in pre- and post-treatment specimens. *P = NS, not significant, by unpaired t-test. Fig. 4: (A) Quantitative PD-L1 score at baseline plotted against survival. (B) Change in quantitative PD-L1 score from pre- to post-treatment plotted against survival. [file 13148_2021_1014_MOESM1_ESM.docx]
